# Recent advances in immunoassay technologies for the detection of human coronavirus infections

**DOI:** 10.3389/fcimb.2022.1040248

**Published:** 2023-01-04

**Authors:** Danqi Wang, Yuejun Chen, Shan Xiang, Huiting Hu, Yujuan Zhan, Ying Yu, Jingwen Zhang, Pian Wu, Fei yue Liu, Tianhan Kai, Ping Ding

**Affiliations:** ^1^ Xiang Ya School of Public Health, Central South University, Changsha, Hunan, China; ^2^ Breast Surgery Department I, Hunan Cancer Hospital, Changsha, Hunan, China; ^3^ Department of Economics and Management, ChangSha University, Changsha, Hunan, China

**Keywords:** human coronavirus, diagnosis, serological testing methods, SARS-CoV-2, immunosensor

## Abstract

Severe acute respiratory syndrome coronavirus-2 (SARS-CoV-2) is the seventh coronavirus (CoV) that has spread in humans and has become a global pandemic since late 2019. Efficient and accurate laboratory diagnostic methods are one of the crucial means to control the development of the current pandemic and to prevent potential future outbreaks. Although real-time reverse transcription-polymerase chain reaction (rRT-PCR) is the preferred laboratory method recommended by the World Health Organization (WHO) for diagnosing and screening SARS-CoV-2 infection, the versatile immunoassays still play an important role for pandemic control. They can be used not only as supplemental tools to identify cases missed by rRT-PCR, but also for first-line screening tests in areas with limited medical resources. Moreover, they are also indispensable tools for retrospective epidemiological surveys and the evaluation of the effectiveness of vaccination. In this review, we summarize the mainstream immunoassay methods for human coronaviruses (HCoVs) and address their benefits, limitations, and applications. Then, technical strategies based on bioinformatics and advanced biosensors were proposed to improve the performance of these methods. Finally, future suggestions and possibilities that can lead to higher sensitivity and specificity are provided for further research.

## 1 Introduction

In late 2019, a novel human coronavirus, severe acute respiratory syndrome coronavirus-2 (SARS-CoV-2), emerged and soon spread worldwide, then developed into a global pandemic within months (Huang et al., 2020; WHO, 2020a). It marks the emergence of the seventh coronavirus that infects humans. The six previously discovered HCoVs include severe acute respiratory syndrome coronavirus (SARS-CoV), Middle East respiratory syndrome coronavirus (MERS-CoV), HCoV-229E (229E), HCoV-OC43 (OC43), HCoV-NL63 (NL63), and HCoV-HKU1 (HKU1) (Su et al., 2016).

**Figure 1:**
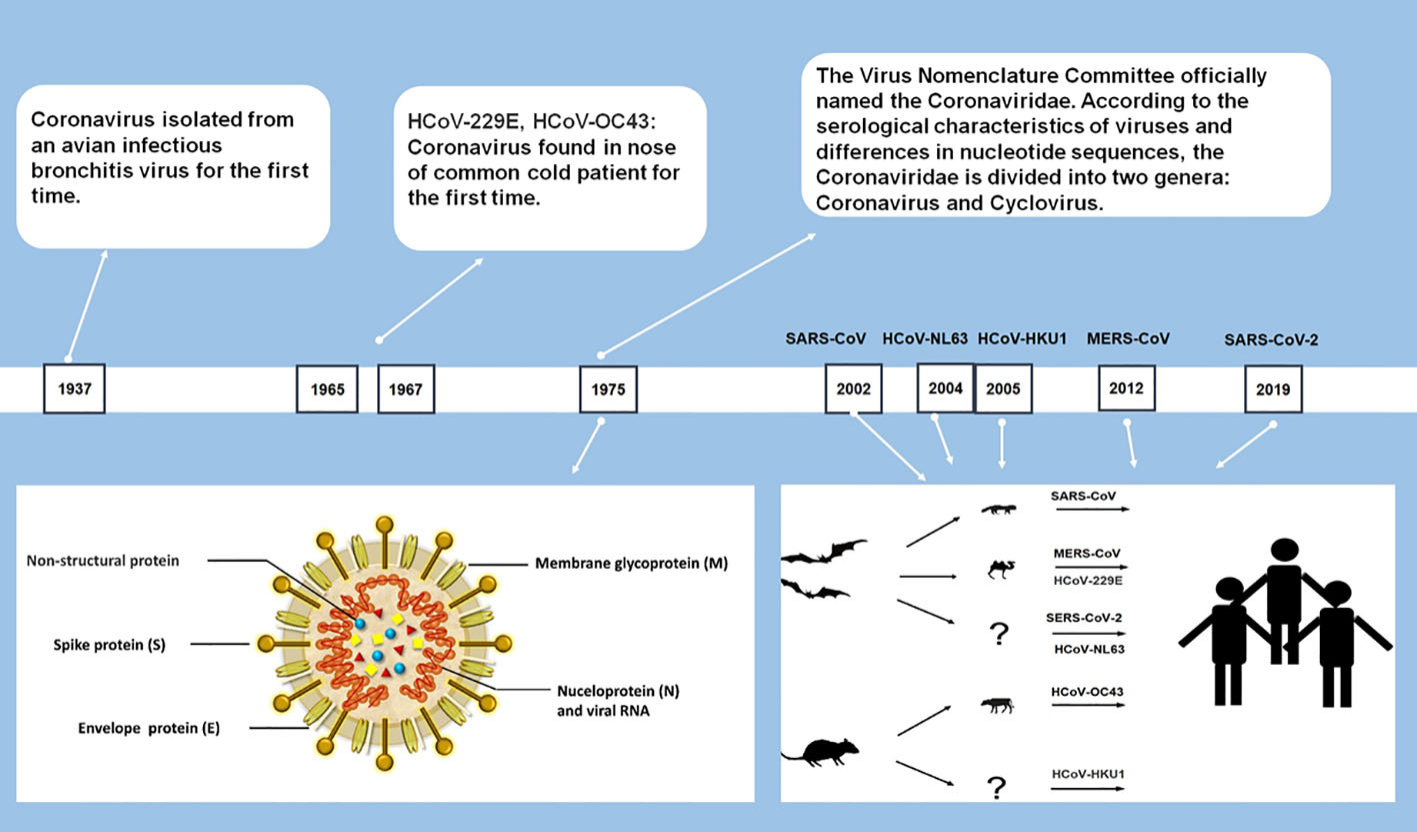
Human Coronavirus: Discovery Timeline, Structure and Origin.

As largest single-stranded RNA viruses with genomes ranging from 26 to 32 kilobases (Chen et al., 2020a), all CoVs are similar in structure and gene expression. Most CoVs contain four structural proteins: spike (S) protein, envelope (E) protein, membrane (M) protein and nucleocapsid (N) protein, and 16 nonstructural proteins. All structural proteins are encoded by the open reading frame (ORF) at the 3’ end, and nonstructural proteins are encoded by ORFs at the 5’ end (**Figure 1**) (Fehr and Perlman, 2015; Neuman and Buchmeier, 2016). CoVs are categorized into four genera: *Alphacoronavirus*, *Betacoronavirus*, *Gammacoronavirus*, and *Deltacoronavirus*. Among the seven currently identified HCoVs, two of them (229E and NL63) belong to *Alphacoronavirus* and the rest (HKU1, OC43, SARS-CoV, MERS-CoV, and SARS-CoV-2) belong to *Betacoronavirus* (Cui et al., 2019; Chen et al., 2020a).

Among the seven HCoVs, 229E, OC43, NL63, and HKU1 mainly infect the upper respiratory tract and causes the common cold with a high incidence in the immunocompetent population (Su et al., 2016). SARS-CoV, MERS-CoV, and SARS-CoV-2 can infect the lower respiratory tract and cause severe respiratory syndrome (Chen et al., 2020a; Huang et al., 2020). During the pandemic in 2003, SARS caused a total of 8,096 infections, approaching about 10% of deaths (Cheng et al., 2007). MERS-CoV has a higher fatality rate than SARS, which caused a total of 2585 cases, including 891 associated deaths (case fatality rate: 35%) globally reported by WHO (WHO, 2022a). Compared with SARS-CoV and MERS-CoV, SARS-CoV-2 shows weaker pathogenicity and stronger transmissibility (Huang et al., 2020; Xu et al., 2020).

The SARS-CoV-2 pandemic has caused substantial morbidity and mortality. As of 16 October 2022, over 621 million confirmed cases and over 6.5 million deaths had been reported globally (WHO, 2022b). Of those infected with SARS-CoV-2, about 40–50% are asymptomatic or mild cases (Mizumoto et al., 2020; Nishiura et al., 2020). However, some of them can be highly contagious (Zou et al., 2020), complicating initial clinical diagnosis and increasing the risk of community transmission. Efficient and accurate laboratory diagnostic methods are one of the crucial means to control the development of the current pandemic and prevent potential future outbreaks. According to the WHO recommendation, real-time reverse transcription-polymerase chain reaction (rRT-PCR) is the preferred laboratory testing method for diagnosing SARS-CoV-2 infection, and immunoassays could be used as supplemental tools (WHO, 2020b). However, with the development of the pandemic, the importance of immunoassay methods has attracted more attention, as it can significantly improve the detection rate when immunoassays and rRT-PCR were conducted simultaneously (Guo et al., 2020; WHO, 2022c). Many immunoassay methods, such as lateral flow immunoassay (LFIA), are not very demanding on experimental conditions and experimenters, and are rapid and easy to use with satisfactory sensitivity and specificity, making them widely applicable to a variety of scenarios. Therefore, in areas where laboratory conditions are unsatisfied, they can be used as the preferred method for first-line screening so that infected individuals can be timely targeted for isolation and treatment. On the other hand, immunoassay methods are important tools to assess seroprevalence and evaluate the effectiveness of vaccination. Given that humans will be coexisting with seven human coronaviruses, including SARS-CoV-2, for a long time and that vaccination will become a routine health protection measure, it is crucial to provide clear evidence of vaccination demands through the serological test. More importantly, more sensitive and rapid detection will be achieved by combining these methods with new functionalized sensing materials and technologies.

Given the versatility of immunoassay methods, it is of great significance for epidemic prevention and control to develop such robust methods and fully explore their value in various application scenarios. Herein, we mainly review the existing immunoassay methods for the detection of HCoVs infections, summarize their advantages and limitations (**Table 1**), and discuss the strategies for improving their performance. This review aims to provide guidance for the further development of immunoassay methods against SARS-CoV-2 as well as other unknown HCoVs that may emerge in the future.

**Table 1:** Overview of mainstream immunoassays for detection of HCoVs infections.

Methods	Applications	Advantages	Limitations
ELISA	Diagnosis in the absence of PCR laboratory conditions; use as supplemental tests with PCR to improve diagnostic accuracy; seroepidemiological survey; evaluation of vaccine efficacy	High throughput; can be automated; semiquantitative or quantitative	Require basic laboratory technician and equipment
WB	Confirmatory test for diagnosis or research	Quantitative determination; multiple target detection in one test	Operation complex; high requirement for experience and equipment; time-consuming
IFA	Confirmatory test for diagnosis or research	High sensitivity and specificity	Operation complex; high requirement for experience and equipment; time-consuming
ICT	POCT; self-testing	Rapid and easy for operation; no requirement for laboratory and technician	Relatively low specificity and sensitivity; high false-negative rate; qualitative determination
Immunosensor	POCT	Portable; rapid and easy for operation; low LOD	Unstable performance; high economic cost

## 2 Immunoassay methods

### 2.1 Enzyme-linked immunosorbent assay

**Figure 2:**
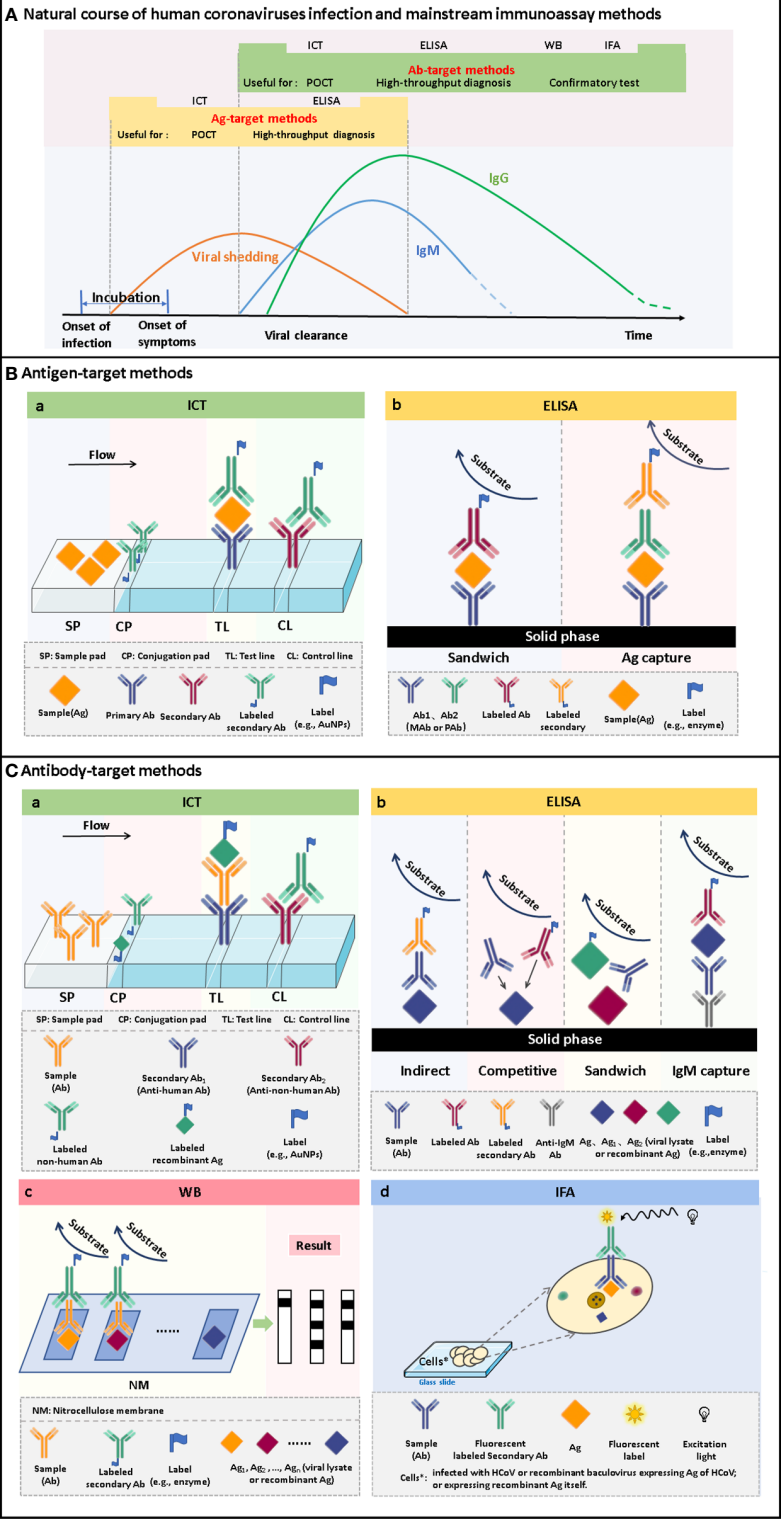
The time windows of viral shedding, and antibody responses against HCoVs infections, and the principles of the mainstream immunoassay methods. **(A)** A typical course of human coronavirus infection. The infection starts with an asymptomatic incubation period. Viral shedding begins during the incubation period and continues for some time after antibody production. Antibodies are produced a few days after infection. Among the antibodies, IgM appears within a few days after infection and can persist for several months. IgG is produced shortly after the appearance of IgM and persists for a longer time. Antigen-target methods, including ICT and ELISA, can be used to screen infected persons during viral shedding. It can realize the differentiation of latent infection and the early diagnosis of disease. After seroconversion occurs, antibody-target methods, including ICT, ELISA, WB and IFA, can be used for diagnosis. It can be used for acute-phase diagnosis as well as epidemiological investigation. **(B)** Schematic of ICT and ELISA for the detection of antigens. **(C)** Schematic of ICT, ELISA, WB and IFA for the detection of antibodies.

Enzyme-linked immunosorbent assay (ELISA) is one of the most widely used immunoassay methods against HCoVs, as well as other viruses, for its ease of use, high throughput, rapid, high sensitivity, and good specificity. It is a quantitative analysis method that shows antigen-antibody reactions in the solid phase through the color change obtained by using an enzyme-linked conjugate and enzyme-substrate to identify the presence of antigens or antibodies in biological fluids (**Figures 2B, C**) (Aydin, 2015). It needs to be performed in a routine laboratory for its requirements of basic equipment and technicians. In areas where equipment is limited, it can also be used for qualitative analysis with naked eyes. A round of ELISA usually takes 2-4 hours and is carried out in batches. Therefore, ELISA is mainly suitable for laboratory diagnosis, research, and epidemiological investigation, and cannot be used for point-of-care testing (POCT).

#### 2.1.1 ELISA for antigen detection

In essence, both the targets of antigen detection and nucleic acid detection are the viral components. In theory, as long as virus shedding occurs, the viral components, whether nucleic acid or protein, can be detected in the corresponding samples (Chen et al., 2004a). Therefore, antigen detection methods can replace nucleic acid detection methods to a certain extent. Researches on coronavirus antigen detection methods are mainly focused on ELISA. Studies evaluated by clinical samples have been summarized in **Table 2**. According to the principle, there are mainly two kinds of antigen detection ELISA: double antibody sandwich ELISA (DAS ELISA) and antigen capture ELISA (principle shown in **Figure 2B**). The antibodies employed are mostly monoclonal antibodies (MAbs) and sometimes polyclonal antibodies (PAbs). Most of these antibodies are produced against the N protein. It mainly due to the fact that the N protein released into the blood is more abundant and earlier than the S protein during viral infection (Che et al., 2004a; Chen et al., 2015; Peng et al., 2020), which makes the N protein easier to be detected.

**Table 2:** ELISA for antigen detection validated by clinical samples.

Virus	Antigen based	Antibody based	Sensitivity(No. positive/no. tested (%))	Specificity(No. negative/no. tested (%))	Comment	Ref
SARS-CoV	Rec N	PAb; MAb	PAb: 17/32 (53.1) in fecal 4/33 (12.1) in urine MAb: 14/32 (43.8) in fecal 5/33 (15.2) in urine	–	–	(Lau et al., 2005)
SARS-CoV	Rec N	MAb	24/24 (100)	197/197 (100)	Coated with a mixture of three different antinucleocapsid MAbs.	(Di et al., 2005)
SARS-CoV	Rec N N2(aa 221-422)N3(aa 249-395)	MAb (coated) pAb (probe)	5-10 dao: 3/4 (75) 15-21 dao: 0/36 (0) 21–90 dao: 0/146 (0)	Health individuals: 103/103 (100) patients with different diseases: 400/400(100)	No cross-reaction with HCoV-OC43, -229E, and -NL63	(He et al., 2005a)
SARS-CoV	N195 (aa 210-423)	MAb	18/18 (100)	60/60 (100)	No cross-reaction with bronchitis virus and chicken coronavirus; detection limit: 37.5pg/mL and 50 TCID50/mL	(Shang et al., 2005)
SARS-CoV	Rec N	MAb	11/13 (84.6)	1272/1253 (98.5)	Linear range:100 pg/mL to 3.2 ng/mL; detection limite: 50 pg/mL; no cross-reaction with other related human and animal coronaviruses.	(Che et al., 2004b)
SARS-CoV	Rec N	MAb	1-5 daos: 80/85 (94); 6-10 daos: 47/60 (78)	824/825 (99.9)	Same method as the above article.	(Che et al., 2004a)
MERS-CoV	Rec N	MAb	–	129/129 (100)	Detection limit: 10 TCID_50_/0.1 mL	(Chen et al., 2015)

Rec, recombinant; N, nucleocapsid protein; Ref, reference; daos, days after onset of symtoms; aa, amino acid position; MAb, monoclonal antibodies; PAb, polyclonal antibody; -,not determined.

For a MAb-based DAS ELISA, the role of each antibody in the MAbs-pair has an important influence on the sensitivity. A pair of MAb, termed P140.19B6 and P140.19C7, which showed specific binding to N protein fragment (amino acids (aa) 141 to 280), were used to develop a hetero-sandwich ELISA to detect the SARS-CoV NP antigen (Das et al., 2010). It showed the highest optical density (OD) value, with a limit of detection (LOD) at the order of magnitude of ng per ml when P140.19B6 was chosen as the capture antibody and P140.19C7 was used as the detecting antibody. However, when the roles of the two antibodies were switched, the OD decreased. This phenomenon may be due to the different affinity for the specific antigen of each MAbs, steric hindrance, or antibody-induced conformational changes in the antigen upon antibody binding (Das et al., 2010).

Appropriately designed and carefully selected MAbs-pairs have also been used to develop ELISAs for detecting other coronaviruses such as MERS-CoV, HCoV-229E, and HCoV-NL63, and these methods all performed well (Sastre et al., 2011; Chen et al., 2015; Li et al., 2019). The MAbs-pairs selected were highly reactive with the C-terminus of the N protein, which showed a high degree of specificity without cross-reaction with other HCoVs. One of the ELISAs for MERS-CoV detection even has a pretty low LOD which is one order of magnitude lower than that of PCR. Inspiringly, the DAS-ELISA established by Sastre et al. could detect HCoV-229E and HCoV-NL63 simultaneously and has the potential for commercialization (Sastre et al., 2011). It uses a MAb that recognizes both the two viruses as a capture antibody, and two MAbs that are specific for HCoV-229E and HCoV-NL63, respectively, as detection antibodies, which makes the assay simple and economical, without compromising sensitivity and specificity.

In 2004, Che et al. established a capture sandwich ELISA to detect SARS-CoV (Che et al., 2004b). The assay employed a mixture of three MAbs specific against N protein for capture and rabbit polyclonal antibodies for detection. When applied to a large number of clinical serum specimens, it exhibited a sensitivity of 96 to 100% at 3 to 5 days after the onset of symptoms, and its specificity was 100% (Di et al., 2005). It suggested that the method is useful for early diagnosis of SARS. The combination of the three different but specific monoclonal capture antibodies played a vital role in ensuring the high sensitivity and specificity of the assay since both degraded and complete virions can bind to the MAbs-coated solid phase. However, coating with too many kinds of antibodies will be counterproductive. When PMAbs were used for coating, the sensitivity to nasopharyngeal aspirates (NPAs) from various stages of the disease course is lower than that of Che’s method, even with a high specificity of 96.7% (Lau et al., 2004).

With the deepening of the research on SARS-CoV-2, antigen-capture ELISA studies based on S protein-specific monoclonal antibodies have been widely followed. For example, Chi et al. (Chi et al., 2020) identified a monoclonal antibody named 4A8 and determined its epitope to be the N-terminal structural domain (NTD) of the S protein. However, Malik et al. (Malik et al., 2021) found that monoclonal antibodies against SARS-CoV cross-reacted with the S protein of SARS-CoV-2 and neutralized its activity. It suggested that the S protein did not exhibit any advantage in increasing sensitivity. Since many of the S protein-based antibody detection methods discussed later in this article have high diagnostic values, it is speculated that the S protein itself should also have diagnostic value as a biomarker, which needs further study.

#### 2.1.2 ELISA for antibody detection

Antibody detection ELISA is a research hotspot of immunoassay methods. A number of ELISAs developed for the detection of antibodies against HcoVs that evaluated by clinical samples have been summarized in **Table 3**. According to the principle, they can be divided into indirect ELISA, double antigens sandwich ELISA, antibody-capture ELISA, and competitive ELISA (**Figure 2C**). Indirect ELISA is the most commonly used. Two main factors influence the performance of the antibody detection ELISA. One is the matching degree between the principle and the target antibody, and the other is the characteristics of the antigen adopted. The antigens applied include the whole virus, the recombinant N protein, the recombinant S protein, the recombinant M protein, recombinant non-structural proteins, and the mixture of several of them.

**Table 3:** ELISA for antibody detection validated by clinical samples.

Virus	Antigen based	Biomarker (Subclass of immunoglobulin)	Sensitivity (No. positive/no. tested(%))	Specificity(No. negative/no. tested (%))	Comment	Ref
SARS-CoV	Whole virus	IgG	54/56(96.4)	204/204(100)		(Shao et al., 2005)
SARS-CoV	Whole virus	IgG	30/36(83.3)	96/96(100)		(Chen et al., 2004b)
SARS-CoV	Whole virus	IgG	117 (100)	813/813 (100)		(Li et al., 2005a)
SARS-CoV	Whole virus	IgM	28/36(77.8)	96/96(100)		(Chen et al., 2004b)
SARS-CoV	Whole virus	Ab(IgG+IgM+IgA)	220/224(98.2)	242/245(98.7)		(Wu et al., 2004a)
SARS-CoV	Rec N	IgG	61/61 (100)	476/483 (98.5)	Rec N expressde by baby hamster kidney cells.	(Haynes et al., 2007)
SARS-CoV	Rec N (aa 1-422)	IgG	10/10 (100)	50/50 (100)		(Lee et al., 2008)
SARS-CoV	Rec N (aa 1-422)	IgG	90/95(94.7)	Not shown.		(Woo et al., 2005b)
SARS-CoV	Rec N	IgG	80/87(92)	-/-(92)		(Saijo et al., 2005)
SARS-CoV	Rec N	IgG	10 daos: 15/16(93.7);20 daos: 16/16 (100);30 daos: 16/16 (100)	131/131(100)		(Timani et al., 2004)
SARS-CoV	Rec N	IgG	146/150(96.2% for late serum samples)	440/450(97.8)		(Chan et al., 2005)
SARS-CoV	Rec N	IgG	6-10daos:12/18(68.4);11-61daos:16/18(89.6)	984/983(99.9)		(Shi et al., 2003)
SARS-CoV	N (aa 1-422)	IgG	100/106(94.3)	142/149(95.3)	IgA: Sensitivity:64/106(60.4) Specificity:144/146(96.6)	(Woo et al., 2004a)
SARS-CoV	N (aa 1-422)	IgG	100/106(94.3)	149/149(100)		(Woo et al., 2004b)
	GST-N	IgG	9/10(90)	–		(Huang et al., 2004)
SARS-CoV	Rec N (aa 110-422)	IgG	10/10 (100)	50/50 (100)		(Lee et al., 2008)
SARS-CoV	NΔ121 (aa 122-422)	IgG	36/36(100) (3 week after onset)	–		(Yu et al., 2007)
SARS-CoV	NΔ121 (aa 122-422)	IgG	36/37(97.3)	175/175(100)		(Yu et al., 2005)
SARS-CoV	Rec N (aa 213-423)	IgG	clinical inpatients:311/442(70.4); convalescent patients:229/302(75.8)	2707/2726(99.3)		(Lu et al., 2005a)
SARS-CoV	Rec N (aa 213-422)	Total Ab	25/35(71.4)	544/544(100)	Double-antigen sandwich ELISA; 229 of 302 (75.8%) samples of convalescent SARS patients were positive	(Chen et al., 2005)
SARS-CoV	N (aa 1-422)	IgM	63/106(59.4)	144/149(96.6)		(Woo et al., 2004a)
SARS-CoV	Rec N (aa 1-422)	IgM	53/95(55.2)	–		(Woo et al., 2005b)
SARS-CoV	NΔ121 (aa 122-422)	IgM	36/36(100)	175/175(100)	MAC-ELISA; Patient’s serum were collected 3 week after onset	(Yu et al., 2007)
SARS-CoV	Rec S (aa 1-1190)	IgG	59/61 (96.7)	480/483 (99.4)	Rec S expressed by HEK-293T/17 cells	(Haynes et al., 2007)
SARS-CoV	Rec S (aa 251- 683) and N (N1, N2, and N3) cocktail	IgG+IgM	18/20(90)	99/100(99)	N1, N2, and N3 comprise almost the whole nucleocapsid protein	(Gimenez et al., 2009)
SARS-CoV	Rec S (aa 250-667)	IgG	56/95(58.9)	146/148(98.6)		(Woo et al., 2005b)
SARS-CoV	Rec S (aa 250-667)	IgM	71/95(74.7)	140/148(93.9)		(Woo et al., 2005b)
SARS-CoV	S1 (aa 540-559)	IgG	9/10(90)	3/6(50)	Synthetic peptides	(Lu et al., 2005b)
SARS-CoV	Rec N	IgG	74/74(100)	209/210(99.5)	aa 111-118 deleted	(Guan et al., 2004a)
SARS-CoV	M	IgG	4/4(100)	–		(Qian et al., 2006)
SARS-CoV	Synthetic peptides (S, M, N)	IgG	69/69(100)	1390 (100)		(Hsueh et al., 2004a)
MERS-CoV	Rec S (aa 318–510)	Ab	62/63(98)	3/3(100)	Rec S expressed by baculovirus expression system; competitive ELISA; dromedary camel sera used as sample.	(Fukushi et al., 2018)
MERS-CoV	Rec S (aa 1-725)	IgG	12/13(92.3)	195/195(100)		
HCoV-229E	N	–	–	16/18(89.9)		(Shao et al., 2007)
HCoV-OC43	Rec N	IgG	10/11(90.9)	39/47(82.9)		(Blanchard et al., 2011)
SARS-CoV-2	S (aa 1-1208)	IgM or IgG	34/40(85)	50/50(100)		(Adams et al., 2020)
SARS-CoV-2	RBD	Total Ab IgM IgG	78/80(97.5) 74/80(92.5) 71/80(88.8)	300/300(100) 300/300 (100) 300/300 (100)	Commercial assay (Wantai)	(Lou et al., 2020)
SARS-CoV-2	S1 subunit	IgG IgA	61/75(81) 73/75(97)	156/157(99) 147/157(94)	Commercial assay (Euroimmun)	(Geurtsvankessel et al., 2020
SARS-CoV-2	N	IgM IgG IgA	188/208(90.4) 162/208(77.9) 194/208(93.3)	285/285(100) 285/285(100) 285/285(100)		(Guo et al., 2020)
SARS-CoV-2	RBD	Ab IgM IgG	161/173(93.1) 143/173(82.7) 112/173(64.7)	211/213(99.1) 210/213(98.6) 195/213(99)		(Zhao et al., 2020)

Rec, recombinant; N, nucleocapsid protein; S, spike protein; Ref, reference; daos, days after onset of symptoms; aa, amino acid position; -,not determined.

Few ELISAs use the whole virus as the antigen reagent because preparing virus particles requires demanding experimental conditions, which is not conducive to mass production and large-scale application. However, for its acceptable sensitivity and specificity (**Table 2**, row 2 to 6), this kind of method can be utilized to investigate the serological profile in the early stage of the epidemic.

The N protein is one of the most abundantly expressed structure proteins in HCoVs and is also a major immunogenic protein (Oliveira et al., 2020; Peng et al., 2020). Its small size and lack of glycosylation sites make it easy to clone and purify efficiently. Therefore, there are a large number of studies on ELISA methods using recombinant N protein as the antigen reagents. The antigens include the intact N protein and all kinds of truncated N proteins. Woo et al. established a series of indirect ELISAs based on recombinant intact N protein (aa 1- 422) for IgG and IgM against SARS-CoV detection (Woo et al., 2004a; Woo et al., 2004b; Woo et al., 2005b). The sensitivity (94.3-94.7%) and specificity (96.6-100%) of IgG detection were at an ideal level, while the sensitivity (59.4%) of IgM was relatively low, though the specificity (96.6%) was at a high level. With appropriate modifications, this N protein-based indirect ELISA was also used to detect IgG and IgM against HCoV-HKU1 (Woo et al., 2005a). A similar difference between the sensitivity of IgG and IgM was also observed when the method was applied to clinical samples (Chan et al., 2005). It suggested that the indirect ELISA is suitable for the detection of IgG but not for IgM. The main reason lies in the higher titers and affinity of IgG, which could strongly compete with IgM for the binding site on the coated antigens (Chen et al., 2004a).

For the detection of IgM, the IgM antibody capture ELISA (MAC ELISA) has a higher sensitivity. A MAC-ELISA based on SARS CoV N 121 protein, an N protein construct with 121 amino acids of the N terminus truncated, showed specificity and sensitivity of 100% (Yu et al., 2007). It could detect the seroconversion of IgM as early as 5 days after disease onset, with a median time of 8 days. The MAC-ELISA captures the serum IgM onto the solid phase with anti-IgM at the first step, and then the solid phase complex reacts with the labeled antigen (**Figure 2C**). This approach prevents IgG and other antibodies with stronger affinity and higher titers from occupying part of the epitopes and reduces the sensitivity. The application of the truncated N protein also contributes to the perfect performance since the operation of truncating can make the specific epitope adequately exposed and the non-specific epitope on the N terminus deleted.

Several HCoVs N proteins share common sequences in the N terminus, which leads to cross-reactivity (Rota et al., 2003). Another study further proved the excellent diagnostic value of the N 121protein. When applied in an indirect IgG ELISA, the non-specific reaction drastically reduced compared to the whole-length N protein-based ELISA (Yu et al., 2005). Moreover, the data obtained by the indirect IgG ELISA based on N 121 strongly indicate the existence of subclinical SARS-CoV infection, although at quite a low rate. It suggests that the rate of missed diagnosis could be reduced by applying this method.

ELISAs based on truncated N proteins are usually more specific than those based on the full-length N protein. However, if the N protein is truncated too short, it may cause the loss of certain specific epitopes, reducing the sensitivity. The recombinant N protein of SARS-CoV derived from aa 213-423, whether applied in an indirect ELISA or a double antigen ELISA, showed a relatively low sensitivity of 70.4~75.8%, albeit with a quite high specificity of 99.3% (Chen et al., 2005; Lu et al., 2005a). It is mainly due to that there are some critical immunodominant epitopes between the aa 122-212 of the N protein. An ideal coating or detection antigen for ELISA should be a protein with all highly-homologous sequences among HCoVs deleted and all immunodominant epitopes retained.

Among the four structural proteins, the S protein has the lowest degree of sequence conservation and contains multiple conformational epitopes (Rota et al., 2003). Therefore, it is a more specific target for serodiagnosis than the N protein (Bosch et al., 2005; Woo et al., 2005c; Li et al., 2020a). Among the newly established methods against SARS-CoV-2, the S-protein-based methods show more satisfactory performance than those against other HCoVs (**Table 3**). In research on the detection of IgG against HCoV-HKU1, an N protein-based and an S protein-based indirect ELISA were performed on the same sera in parallel. It was found that 28.5% (6/21) of N protein-based seropositive samples, confirmed as negative by western blot, were tested negative by S protein-based assay (Chan et al., 2009). The S protein-based ELISA is more specific and could minimize the cross-reactivity. However, as shown in **Table 3**, the sensitivity of these S protein-based ELISAs varies widely (58.9-98%). The underlying cause for the difference is the different preparation methods of the recombinant S protein. The methods based on S proteins expressed by the eukaryotic system showed a high level of sensitivity (Haynes et al., 2007; Fukushi et al., 2018), while those based on synthetic peptides or S proteins expressed by the prokaryotic system showed a low sensitivity (Lu et al., 2005b; Woo et al., 2005b; Gimenez et al., 2009). The post-translational modifications in the eukaryotic expression system can ensure the correct expression of conformational epitopes. In contrast, synthetic peptides or proteins expressed by the prokaryotic system can only display some linear epitopes that could not adequately represent the native antigenicity of the S protein. Although ELISA based on the full-length S protein has satisfactory performance, considering its large molecular weight and complicated conformation, it is usually challenging to prepare soluble full-length S protein, which is not conducive to mass production. Therefore, S protein fragments that can be prepared as soluble proteins are practically required. Since the S1 subunit, compared with the S2 subunit, contains more epitopes and is more poorly conserved in the seven HCoVs (Maache et al., 2006; Premkumar et al., 2020), it would have a high diagnostic value to establish ELISA using the S1 subunit or its fragments (Okba et al., 2019; Freeman et al., 2020). More importantly, specific fragments derived from the S1 subunit have an advantage over the N protein in early diagnosis. Whether applied to indirect ELISA or MAC ELISA, the IgM detection ELISA based on the S protein was more sensitive than that based on the N protein (Woo et al., 2005b; Liu et al., 2020). When the receptor binding domain (RBD) was applied in MAC ELISA for detection of IgM against SARS-CoV-2, it even showed higher sensitivity than the DAS ELISA based on the same antigen for detection of IgG (Lou et al., 2020).

One of the critical roles of the M protein is inducing virus neutralization, which makes it an attractive target for developing vaccines, drugs, and diagnostic reagents (Ujike and Taguchi, 2015; Jorrissen et al., 2021). Two recombinant M proteins, M fusion protein (aa 1-43) and cytoplasmic-domain (aa 138-222) of the M protein, have been applied to develop indirect ELISA to validate their potential diagnostic value for SARS (Han et al., 2004; Carattoli et al., 2005; Qian et al., 2006). All of them were specifically reacted with sera from SARS-CoV positive patients, confirmed by western blot or immunocytochemical assay, even when the sera were diluted to 1:12,800. However, these researches were all tested with a few, 4 or 6, confirmed positive sera. It requires confirmation with more positive samples to validate the diagnostic value of M protein.

Another option to improve the performance is coating the microplate with multiple antigens. An ELISA for the detection of IgG-plus-IgM against SARS-CoV used a cocktail of four recombinant polypeptides as coating antigens. The cocktail includes a recombinant S-protein (aa 251-683) and three recombinant N-protein that comprise almost the whole nucleocapsid protein. The method showed 99% specificity and 90% sensitivity (Gimenez et al., 2009). The false-negative results may be due to that the recombinant S protein is too short to cover all the epitopes. A mixture of site-specific synthetic peptides taken from the S, M, and N proteins of SARS-CoV, when employed in an IgG detection ELISA, showed a rate of 100% both for specificity and sensitivity. And it even could recognize asymptomatic infected individuals (Hsueh et al.,2004a). It suggested that carefully selected peptides with the amino acid sequence homologous to other HCoVs deleted help to reduce the cross-reaction ratio, and the mixture of these peptides helps to capture antibodies elicited by different epitopes, which will improve the sensitivity.

### 2.2 Western blot

Western blot (WB) assays have been routinely used as confirmatory tests for the diagnosis of several viral infections, such as those caused by the hepatitis C virus, human immunodeficiency virus, and human T-cell lymphotropic virus. Thus, this platform also has a potential value to be used for confirmatory diagnosis of HCoVs infections. Conventional WB uses virus lysate as the antigen reagent. The lysate is separated into multiple purified protein bands by gel electrophoresis and then reacts with the serum to detect the presence of the corresponding antibodies (**Figure 2C**). This kind of method needs to be implemented in a BSL-3 laboratory. In order to seek safer approaches, most of the WB methods for antibody detection turned to employ recombinant proteins as antigen reagents. Considering the complicated procedures of WB and its strict requirements for technicians, even the modified WB is not suitable for first-line detection. A detailed summary of studies applying WB for the detection of antibodies against HCoVs validated by clinical samples is listed in **Table 4**.

**Table 4:** WB for antibody detection validated by clinical samples.

Virus	Antigen based	Biomarker (Subclass of immunoglobulin)	Sensitivity(No. positive/no. tested(%))	Specificity(No. negative/no. tested(%))	Comment	Ref
SARS-CoV	Rec N Rec S (aa 511-1255)	IgG IgG	39/40(97.5) 19/40(47.5)	5/5(100) 5/5(100)		(Huang et al., 2004)
SARS-CoV	N195 ( aa 228-423)	IgG+lgM IgG lgM	40/44(90.9) 39/44(88.6) 25/44(56.8)	226/230(98.3)	No cross-reaction with chicken, pig, and canine coronaviruses;	(He et al., 2004a)
SARS-CoV	Rec GST-N(aa 121-422) & viral lysate antigens	IgG	40/40(100)	150/150(100)	Different antigen proteins were arrayed on the same strip.	(Ming et al., 2004)
SARS-CoV	Rec N & Rec M & Rec S5 (aa 678-888) & Rec S6 (aa 884-1113)	IgG IgA IgG or IgA	34/46(73.9) 41/46(89.1) 42/46(91.3)	43/44(97.7) 39/44(88.6) 39/44(88.6)	The sample pool contains serum collected from acute phase as well as convalescent phase.	(Wu et al., 2004a)
SARS-CoV	Rec N	IgG	34/34(100)	98/100(98%)	Serum samples from healthy donors used as negative control, which showed strong reactivity to the nucleocapsid proteins of HCoV-229E and HCoV-OC43, with positive results in 97% and 99%, respectively.	(Che et al., 2005)
SARS-CoV	Rec N Rec S1 (aa 14-760) Rec S2 (aa 761-1190)	IgG	30/30(100) 30/30(100) 26/30(86.6)	0/48(0) 48/48(100) 48/48(100)	Serum samples from healthy donors as negative control.	(Maache et al., 2006)
SARS-CoV	Rec N (aa 120-422) Rec U274 (aa 134–274)	IgG IgM IgA IgG IgM IgA	CP: 81/81(100) AP: 2/7(28.6) AP: 7/7(100) AP: 7/7(100) CP: 59/81(73) AP: 0/7 AP: 1/7 AP: 4/7	100/100(100) 100/100(100)		(Tan et al., 2004)
SARS-CoV	S1 subunit S2 subunit	IgG	15/20(75) 17/20(85)	34/40(85) 40/40(100)	Rec S were expressed in an *E. coli* system.	(Wang et al., 2004)
SARS-CoV	Rec N Rec S1(aa 14–403) Rec S2 (aa 370–770) Rec S3 (aa 738–1196)	IgG	10/10(100) 5/10(50) 3/10(30) 7/10(70)	–	Rec S were expressed in an *E. coli* system.	(Wang et al., 2005)
SARS-CoV HCoV-229E HCoV- OC43	Rec N Rec N Rec N	IgG IgG IgG	CP: 49/49(100) AP: 4/6(66.7) CP: 6/6(100) AP: 5/6(83.3) CP: 6/6(100)	25/25(100) - - - -		(Lehmann et al., 2008)
HCoV-229E	Rec N (aa 9-389) & Rec S (aa 54-1173)	IgG	10/10(100)	1/1(100)	A neonatal control serum.	(Pohl-Koppe et al., 1995)

Rec, recombinant; N, nucleocapsid protein; S, spike protein; Ref, reference; daos, days after onset of symptoms; aa, amino acid position; -,not determined; AP, acute phase; CP, convalescent phase.

Similar to ELISA, the recombinant proteins applied in WB are mainly N protein, followed by S protein (**Table 4**). For N protein-based WB, the sensitivities (90.9~100%) of the full-length N protein and truncated N protein are almost at the same level when testing convalescent serum samples. However, their specificities are significantly different, ranging from 0 to 100%. Such a large range is closely related to the selection of the N-protein fragment. In the most extreme research case, the method based on full-length N protein for the detection of antibodies to SARS-CoV established by Maache et al. produced a 100% false-positive rate when 48 serum samples collected from healthy donors were tested (Maache et al., 2006). This situation is mainly caused by the cross-reaction of the healthy donor serum samples with the four other HCoVs, HCoV-NL63, -OC43, -HKU1, and -229E, with which the general population was widely infected (Che et al., 2005). In order to reduce cross-reactivity, it is necessary to develop WB methods employing recombinant N protein with highly-homologous sequences of HCoVs deleted. As found in ELISA, WB based on recombinant N proteins with about 100-200 amino acids at the N-terminus deleted have high specificity (Guan et al., 2004b; He et al., 2004a; Tan et al., 2004). In contrast, all the WB based on S proteins, whether full-length or truncated, have high specificity, while the sensitivity varies depending on the recombinant protein amino acid sequence and the stage of the disease course (Huang et al., 2004; Jiang et al., 2021). Since the amino acid sequence homology of the S protein among HCoVs is lower than that of the N protein (Rota et al., 2003), the S protein-based methods have a lower cross-reaction rate, which is beneficial to improve the specificity of the method. With WB based on specific fragments derived from S protein (Wu et al., 2004a), SARS patients could be identified within the first week, as early as 2-3 days, after symptom onset. It suggested that the S protein has a potential value for early diagnosis.

However, a WB method based on a single protein could give only limited information similar to that provided by an ELISA or an IFA (Guan et al., 2004b). The advantage of WB as a confirmatory test should be that, with an array of immobilized antigen proteins, it can simultaneously detect antibodies against several different antigenic components of an HCoV. Thus, it can give information that other immunoassay methods lack but are needed for precise serological differentiation and confirmation. On the basis of the conventional WB using SARS-CoV lysate as the antigen reagent, Guan et al. added a recombinant N protein, with a conserved motif found in other CoVs deleted, for confirmatory serodiagnosis of SARS (Guan et al., 2004b). By setting proper criteria, the assay could differentiate SARS patients from healthy donors or non-SARS patients with 100% accuracy. Moreover, it could also identify false-positive results produced by ELISA through band patterns different from those of true-positive samples from SARS patients. It fully demonstrated the advantages of the multiple proteins based WB as a confirmatory test. However, if the protein components derived from the virus lysate were replaced with recombinant proteins, a safer and more popular way could be provided. Wu et al. established a WB using four recombinant proteins N, M, S5, and S6 as antigens for early detection of IgG and IgA against SARS CoV (Wu et al., 2004a). Using the WB along with RT-PCR, it can significantly improve the confirmation rate by 24.1%, from 48.1% (RT-PCR alone) to 72.2%. Moreover, IgA antibodies against these four proteins are of great value for diagnosing infection within one week after illness onset. Other WB assay platforms based on multiple proteins mainly use specific fragments of N protein and S protein as antigens simultaneously (Woo et al., 2004d; Maache et al., 2006). The role of the N protein is to ensure the sensitivity of the method for its high expression level, while the S protein is to improve the specificity for its low sequence homology among all coronaviruses. Although some studies have attempted to add M protein or U274, a unique protein of SARS-CoV, to the multi-protein combinations, it seems that these two proteins have a pretty low contribution rate to the improvement of detection performance (Tan et al., 2004; Wu et al., 2004a). Therefore, the value of antigen proteins, other than S protein and N protein, in the establishment of multi-protein-based WB methods remains to be explored. In general, by selecting an ELISA as the initial screening method and then using the above-mentioned WB assay platforms to confirm the positive samples, false positive results can be effectively excluded.

It is worth mentioning that WB has another advantage. It can detect antibodies against multiple viruses with a single strip simultaneously. Such an assay was developed by Lehmann et al. based on a strip coated with recombinant N proteins of all known HCoVs (Lehmann et al., 2008). Screening for IgG in convalescent sera reached 100% sensitivity. Therefore, it is useful in large-scale epidemiologic studies. Replacing the secondary anti-human IgG with anti-human IgM can easily adjust the assay to detect IgM, which might be favorable for diagnosing acute infections.

### 2.3 Immunofluorescence assay

Immunofluorescence assay (IFA) has a high value for the application as a confirmatory test since its high level of sensitivity and specificity (Andrey et al., 2020; Meyer et al., 2020). The high sensitivity comes from the high-efficiency signal amplification ability of the fluorescent label. The specificity is attributed to its effective reduction of false-positive reactivities through the localization of antigen-antibody reactions in cells. The direct IFA for antigen detection requires virus cultivation, which needs to be completed in a BSL-3 laboratory, so little research was focused on this aspect. It is safer and more convenient to employ recombinant protein-based indirect IFA to detect antibodies. Methods validated by clinical samples have been summarized in **Table 5**. The principle, in brief, is that a fluorescent-labeled secondary antibody is used to detect the antibody-antigen reaction between the antibody from the serum specimen and the virus or antigen protein contained in the cells seeded and fixed on the glass slide (**Figure 2C**). Compared with WB, IFA has better performance, but it has higher requirements for technical experience, while inappropriate operation has a great impact on the accuracy of the results. Therefore, IFA is more commonly used as confirmatory test in scientific research, rather than in clinical diagnosis.

**Table 5:** IFA for antibody detection validated by clinical samples.

Virus	Antigen based	Biomarker (Subclass of immunoglobulin)	Sensitivity (No.positive/no. tested(%))	Specificity(No.negative/no. tested (%))	Comment	Ref
SARS-CoV	SARS-CoV-infected Vero E6 cells	IgG IgM	(222/252)99.1	(215/217)87.8		(Wu et al., 2004a)
SARS-CoV	RecN	IgG	(45/46)97.8	100		(Zhu et al., 2005)
SARS-CoV	RecS (aa 441-700)		(21/21)100	(142/142)100		(Manopo et al., 2005)
SARS-CoV	N195-Sc fusion protein	IgG IgM	IgG:(22/23)95.6 IgM: lower	IgG:(64/64)100 IgM: 100	N195(aa 228-423); Sc: (aa 441-700)	(He et al., 2005b)
SARS-CoV	Rec S	IgG	CP: 74/74(100); AP(2 to 9 daos): 0/74(0)	100/100(100)		(Tan et al., 2004)
HCoV-HKU1	Rec S	IgG		(53/100)53		(Zhou et al., 2010)
SARS-CoV-2	Rec S	IgG	165/181(91.2)	320/326(98.2)		(Meyer et al., 2020)

Rec, recombinant; N, nucleocapsid protein; S, spike protein; Ref, reference; daos, days after onset of symptoms; aa, amino acid position; -,not determined; AP, acute phase; CP, convalescent phase.

There are two ways to express recombinant proteins for IFA. One is to use baculovirus expressing recombinant protein to infect insect cells, and the other is to transfect cells with recombinant protein expression plasmid. Both the two ways could be carried out in the BSL-2 laboratory. In these two ways, full-length as well as truncated S and N proteins have all been used to establish IFAs (He et al., 2004a; Manopo et al., 2005; Zhu et al., 2005; Zhou et al., 2010; Andrey et al., 2020; Meyer et al., 2020). In indirect IFA, eukaryotic cells are used as the expression system for recombinant proteins, which can effectively ensure the proper folding and glycosylation of the S protein (Tan et al., 2004). Therefore, this method can detect antibodies against the large S protein more accurately. Manpo established an indirect IFA based on a truncated S protein (aa 441-700) of SARS-CoV (Manopo et al., 2005). It gave a specificity and sensitivity of 100% when assessed with a panel of clinical samples collected in 7-76 days post infection, showing no cross-reactivity and was compatible with whole virus-based IFAs. A seroepidemiological survey of the general asymptomatic population also showed that the S-based indirect IFA enabled specific detection of IgG or IgM to the four non-severe acute respiratory syndrome HCoVs, OC43, 229E, HKU1, and NL63, individually (Zhou et al., 2013). In the course of HCoVs infection, antibodies against S protein usually appear and disappear later than that against N protein (Zhou et al., 2010). In theory, detecting antibodies against S protein alone is not conducive to early diagnosis of infection to some extent. Therefore, the joint detection of antibodies against N protein and S protein is necessary to improve the detection efficiency. He et al. established a novel indirect IFA based on the recombinant N195-Sc fusion protein for the diagnosis of SARS-CoV infection (He et al., 2005b). The N195 protein (He et al., 2004a) and Sc protein (Lu et al., 2004; Manopo et al., 2005) had been identified as major immunodominant fragments of the N protein and S protein, respectively. The method showed a sensitivity of 97% and a specificity of 100%. The detection rate was completely identical to the two conventional indirect IFAs based on whole-virus. Compared with indirect IFA based on N195 alone, the novel one showed higher IgM detection levels and stronger positive signals. It means that this method is reliable for early diagnosis since IgM is the primary indicator of the acute phase. Although indirect IFA based on recombinant protein is safer and more convenient, the observation and interpretation of results still require experienced laboratory technicians to ensure quality. Therefore, it can only be used as a confirmatory test rather than a first-line screening test.

### 2.4 Immunochromatography test

Immunochromatography test (ICT), also known as lateral flow immunoassay (LFIA), is a technique that combines immunoassay and chromatography on a nitrocellulose membrane immobilized with capturing reagents at the test and control zones (**Figures 2B, C**). Under the capillary force, the added liquid sample will flow along the membrane. In the process of liquid flow, the analytes first bind to the antigens or antibodies labeled with report particles, such as gold nanoparticles (AuNPs), and then to the capturing reagents, forming colored lines. The result can be judged by naked eyes (Koczula and Gallotta, 2016). ICT is an ideal POCT platform for the detection of antibodies as well as antigens. The advantages are short time consuming (15-30 minutes), easy operation, no specialized training required, no equipment needed, durable stability, and low cost, which makes it especially suitable for resource-limited settings and areas (Kim et al., 2019a). Although ICT is widely used in the diagnosis of various infectious diseases (Koczula and Gallotta, 2016; Kim et al., 2019a) and commercial kits for the detection of HCoVs are emerging one after another, their performances are usually less than satisfactory.

As shown in **Table 6**, the specificities of these methods are at the same level, but there is a very significant difference in sensitivities (**Table 6**). A moderately high sensitivity means the existence of a certain amount of false-negative results, which is a common problem in the applications of ICT, even for the diagnosis of other viruses (Chan et al., 2013). The sensitivity of these ICT methods is mainly limited by the *K_d_* (dissociation constant) of the antibody-antigen reaction and the signal-amplification capability of the signal labels. Compared to methods such as ELISA, conventional ICT, which uses colloidal gold as the signal lable, requires a higher analyte load to produce a visible line to the naked eye (Koczula and Gallotta, 2016). In order to overcome these limitations, it is recommended to introduce new labeling methods, such as fluorescence (Thuy Tien et al., 2018), nanosphere (Chen et al., 2020b), and quantum dots (QDs) (Lu et al., 2019), when developing new ICT methods. Considering the high false-negative rate of ICT, it is more suitable for rapid screening of suspected cases. The negative results should be further confirmed by other more reliable methods.

**Table 6:** ICT for antibody detection validated by clinical samples.

Virus	Biomaker	Antigen based	Antibody based (MAb or PAb)	Sensitivity (No. positive/no. tested(%))	Specificity(No. negative/no. tested(%))	Comment	Ref
SARS-CoV	IgG	Rec N		44/131 (33.6)	111/113 (98.2)		(Wu et al., 2004a)
SARS-CoV	IgG	Rec N		42/42 (100)	209/210 (99)	Rec N with aa 111-118 deleted	(Guan et al., 2004a)
SARS-CoV	N	N (aa 244-260)	MAb	–	150/150 (100)	Detection limite: 7.87× 10^2^ TCID_50_/mL of SARS-CoV	(Kogaki et al., 2005)
MERS-CoV	N	Rec N (aa 10-413)	MAb	62/66 (93.9%)	18/18 (100%)	Camel nasal swabs as sample	(Song et al., 2015)
MERS-CoV	N	Rec N	MAb	13/16 (81%)	65/65 (100%)	Dromedary nasalswabs as sample	(Chen et al., 2016)
SARS-CoV-2	IgG & IgM	Rec S & N		101/113(89)	97/98(99)	Commercial assay (Cellex)	(Geurtsvankessel et al., 2020)
SARS-CoV-2	IgG & IgM	Rec S & N		113/113(100)	87/98(88)	Commercial assay (Orient/Healgen)	(Geurtsvankessel et al., 2020)
SARS-CoV-2	IgG & IgM	Rec N		102/113(90)	83/98(85)	Commercial assay (Intec)	(Geurtsvankessel et al., 2020)
SARS-CoV-2	IgM or IgG	synthetic S, M, and N proteins		69/97(71.1)	51/53(96.2)		(Shen et al., 2020)

Rec, recombinant; N, nucleocapsid protein; Ref, reference; aa, amino acid position; -,not determined; MAb, monoclonal antibodies.

### 2.5 Newly developed immunosensor

Traditional immunoassays are usually time-consuming, require qualified technicians and relatively sophisticated instrumentation. In contrast, immunosensors are miniaturized integrated devices that allow rapid, easy-to-use, and point-of-care detection. A prominent feature of immunosensors is their extremely high sensitivity, which is usually accompanied by poor stability. With the maturity of the technology in related fields, this problem is gradually improving. Herein, we mainly focus on two kinds of well-developed immunosensor, optical immunosensor and electrochemical immunosensor, which has mushroomed during the pandemic of COVID-19.

#### 2.5.1 Optical immunosensor

Optical immunosensor is a combination of optical technology and biosensor. It monitors optical signals caused by antigen-antibody reactions. It has many advantages, such as real-time readout, specificity, biocompatibility, low LOD, and high sensitivity (Vermisoglou et al., 2020).

**Figure 3:**
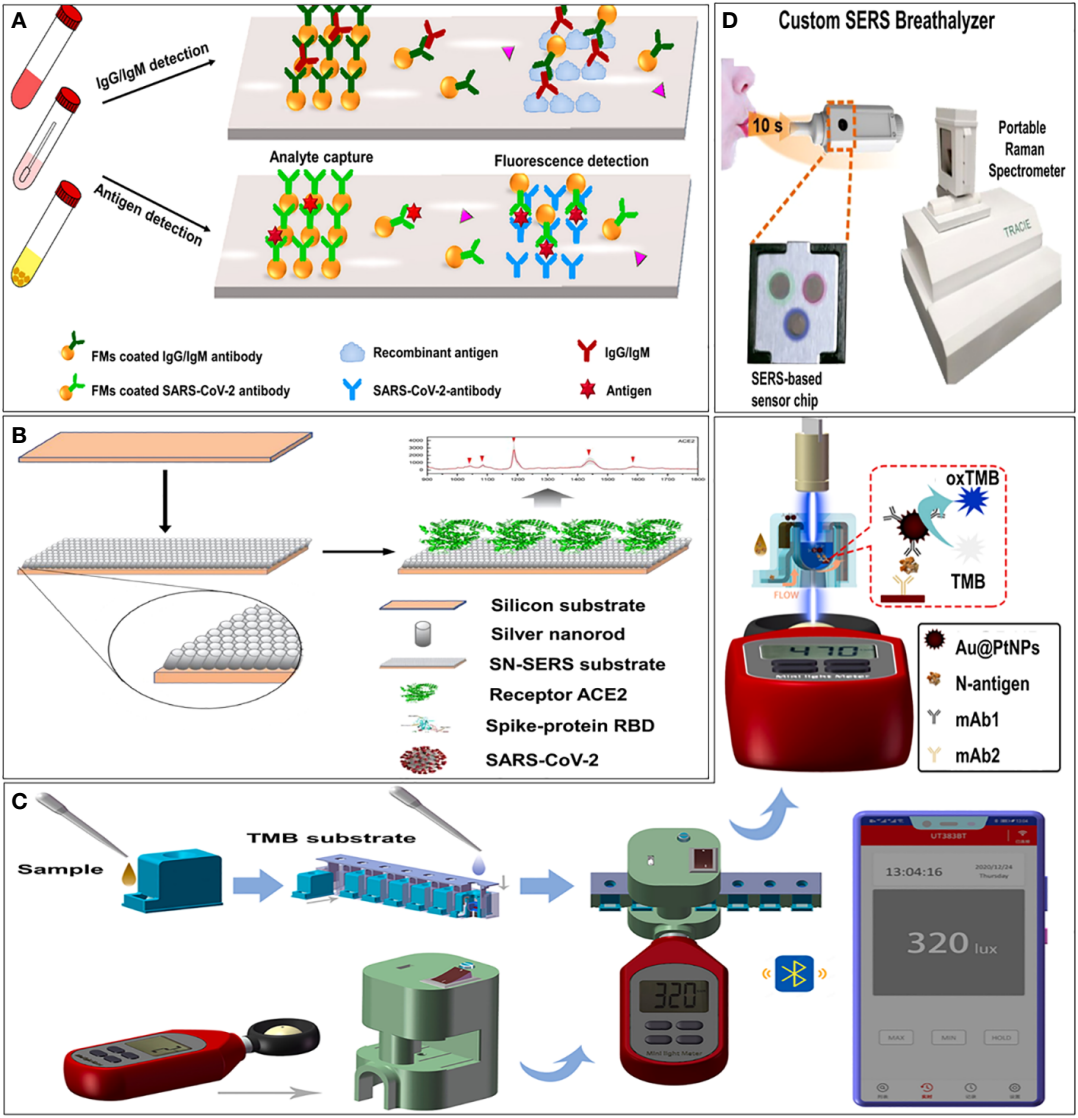
Examples of optical immunosensors. **(A)** Schematic illustration of the microfluidic fluorescence immunoassay for IgG/IgM/antigen detection of SARS-CoV-2 (Lin et al., 2020). **(B)** Schematic illustration of principle of immunoreaction, optical signal transmission and photometer sensing (Zhang et al., 2021). **(C)** Schematic illustration of procedure of smartphone-based NLICS (Liang et al., 2021a). **(D)** Schematic illustration of overview of our SERS-based strategy to identify COVID-positive individuals using their breath volatile organic compounds (BVOCs) (Leong et al., 2022).

Immunosensensor based on fluorescence signals is one of the most sensitive biosensors. Lin et al. established a portable microfluidic immunoassay system (Lin et al., 2020), which integrating a homemade fluorescence detection analyzer, SARS-CoV-2 diagnostic microchips, and multiple immunoassays for detecting three biomarkers (IgG, IgM, and antigen). The platform achieved POCT within 15 minutes, demonstrating easy to use, rapid, portable, and highly sensitive (**Figure 3A**). The sensor incorporates an RBD binder which ensures a LOD of 15 pM and the minimal signal is 50 fold over the background (Quijano-Rubio et al., 2021). By coupling localized surface plasmon and fluorescence techniques, Huang et al. developed a localized surface plasmon coupled fluorescence (LSPCF) fiber-optic biosensors to detect SARS-CoV (Huang et al., 2009). The LOD of the LSPCF fiber-optic biosensor was at least 104 folds of ELISA, which has improved the LOD of SARS-CoV N protein to 0.1 pg/mL in serum.

Among the coupling technologies between smartphones and sensors, the docking of optical sensors is the most mature. A smartphone-based nano-enzyme-linked immunochromatographic sensor (NLICS) has been reported. The sensor was developed for quantitative detection of the SARS-CoV-2 N protein. It integrated the optical sensor and disposable ICT strips with a smartphone (**Figure 3C**). Immunoreaction and enzyme-catalyzed reactions were carried out on the ICT strip, and the photometer in the smartphone was used to read the optical signal through a biosensor channel (Liang et al., 2021). NLICS has a low LOD, with a linear detection range between 0.05 and 1.6 ng/mL, which was more sensitive than conventional ICT. The method can be potentially used for POCT.

Surface-enhanced Raman scattering (SERS) is known as an ultra-sensitive molecular spectroscopy technique. It shows great potential in *in vitro* analysis, such as body fluids, due to its high sensitivity and high resistance to interference from sample matrices (Hang et al., 2022). Zhang et al. (Zhang et al., 2021) functionalized an aligned silver-nanorod SERS array with cellular receptor angiotensin-converting enzyme 2 (ACE2) in oblique angle deposition. ACE2 was used as an anchor to capture SARS-CoV-2 from samples. It has successfully quenched SERS signal intensively in the presence of SARS-CoV-2 spike proteins (**Figure 3B**). Leong et al. (Leong et al., 2022) design a SERS-based breathalyzer to distinguish breath volatile organic compounds profiles of SARS-CoV-2 positive individuals, which can quickly identify infected individuals in a short time (**Figure 3D**).

#### 2.5.2 Electrochemical immunosensor

An electrochemical biosensor is usually composed of an electrode functionalized with conductive materials and immobilized antibody or antigen, which can generate an electrochemical signal in response to specific target binding. It is an attractive option because of its potential for miniaturization.

Yakoh et al. have developed a specific and sensitive immunosensor for detecting antibodies against SARS-CoV-2 (Yakoh et al., 2021). The sensor was based on a label-free paper-based electrochemical platform. The presence of the target antibody would interrupt the redox conversion of the redox indicator, resulting in a decreased current response. In order to demonstrate the practicality of this electrochemical paper-based analytical device (ePAD), 17 clinical serum samples were tested with the prepared ePAD. The sensitivity and specificity were calculated to be 100% and 90%, respectively.

**Figure 4:**
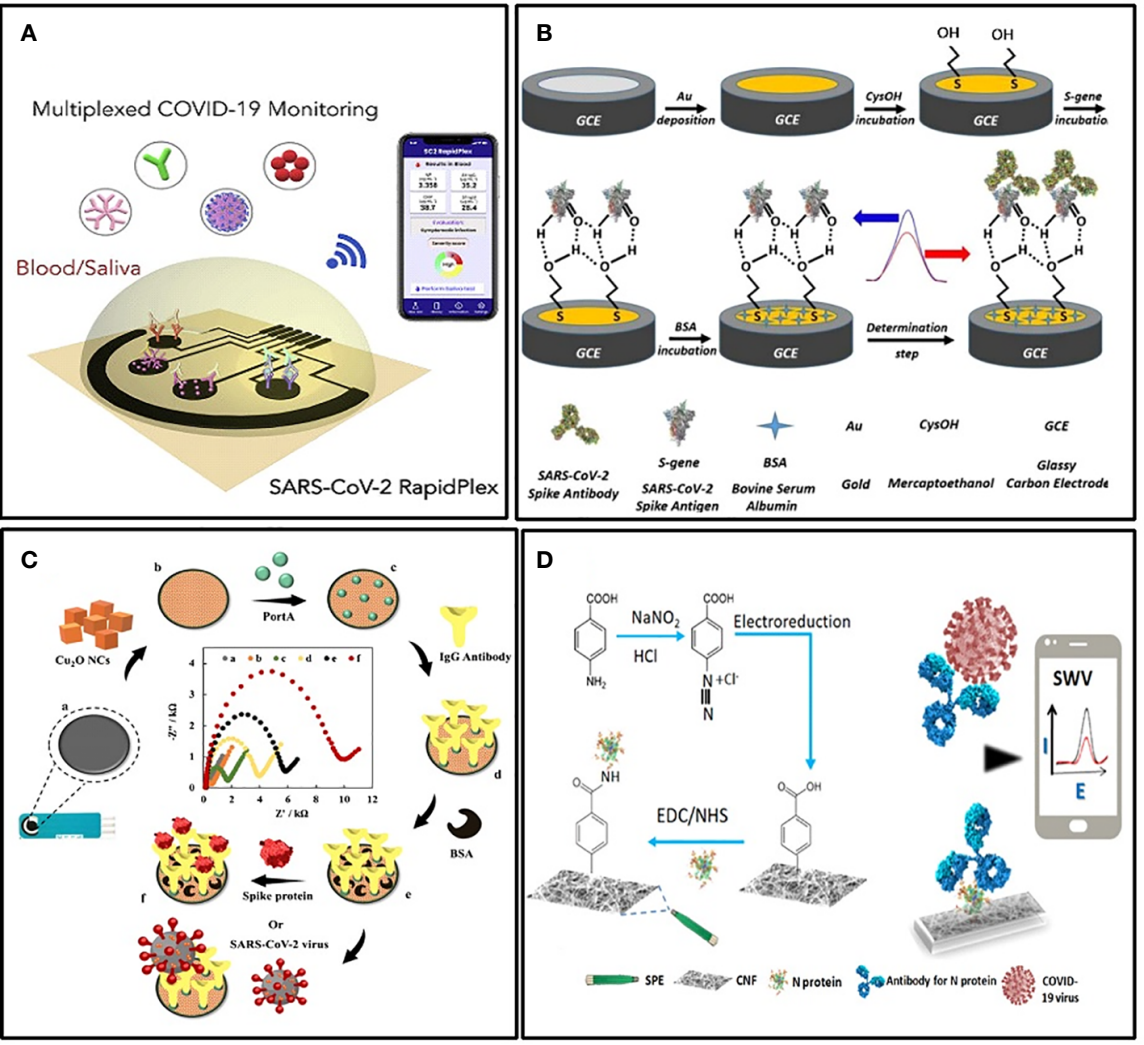
Examples of electrochemical biosensors. **(A)** Schematic illustration of the wireless graphene-based telemedicine platform for rapid and multiplex electrochemical detection of SARS-CoV-2 in blood and saliva (Torrente-Rodrıguez et al., 2020). **(B)** Schematic illustration of preparing BSA/S-gene/CysOH/Au/GCE for the electrochemical detection of the SARS CoV-2 spike antibody (Liv et al., 2022). **(C)** Schematic illustration of the electrochemical immunosensor with Cu2O nanocube coating for detection of SARS-CoV-2 spike protein (Rahmati et al., 2021). **(D)** Schematic illustration of the cotton-tipped electrochemical immunosensor for COVID-19 (Eissa and Zourob, 2021).

Electrochemical immunosensors targeting S protein or its specific antibody generally performed well. Liv et al. developed an electrochemical biosensing platform functionalized with gold clusters and the S protein of SARS-CoV-2 (Liv et al., 2022) (**Figure 4B**). It could detect as low as 0.03 fg/mL of target antibody in synthetic media and spiked saliva or oropharyngeal swab samples within 35 minutes. The sensitivity was approximately 109 times more than LFIA. For sensitive detection of the S protein of SARS-CoV-2, Rahmati et al. modified the disposable screen-printed carbon electrode (SPCE) with Cu_2_O nanocubes and immobilized the S-specific IgG onto the electrode surface in an ordered orientation through staphylococcal protein A (**Figure 4C**) (Rahmati et al., 2021). The LOD also achieved 0.04 fg/mL without any cross-reactivity. For POCT, this kind of sensor is very practical.

Another form of POCT sensor that facilitates mass screening is integrating the sampling and testing process into a single tool. For this purpose, Eissa et al. integrated the sample collection and detection tools into a single platform by coating screen-printed electrodes with absorbing cotton padding (Eissa and Zourob, 2021) (**Figure 4D**). The detection was achieved simply by swabbing and a competitive assay with the N protein-specific antibody in the solution. The LOD was 0.8 pg/mL, and there was no significant cross-reactivity with other HCoVs. In addition, the detection of multiple targets is also meaningful in improving the detection rate. Rebeca M et al. demonstrated a multiplexed, portable, wireless electrochemical platform, the SARS-CoV-2 RapidPlex, for ultra-rapid detection (Torrente-Rodríguez et al., 2020). It can simultaneously detect N protein, IgM, IgG, and the inflammatory biomarker C-reactive protein, using their mass-producible laser-engraved graphene electrodes (**Figure 4A**).

#### 2.5.3 Simultaneous detection of human coronaviruses

In view of the high infection rate of the other four non-sever HCoVs in the population, the simultaneous detection of antibodies to multiple coronaviruses is of great significance for the seroepidemiological survey as well as for rapid and accurate screening of pathogens. Trivedi et al. developed and evaluated a multiplexed magnetic microsphere immunoassay (MMIA) to simultaneously detect IgG antibodies specific to recombinant N proteins from HCoV-229E, HCoV-NL63, HCoV-OC43, HCoV-HKU1, SARS-CoV, and MERS-CoV (Trivedi et al., 2019). MMIA allows for higher sample throughput with less reagent, labor, sample, and material costs, and without compromising sensitivity and specificity. Thus, it is feasible to be used in large scale seroprevalence studies. Besides, protein microarray harbors proteins from different HCoVs is also a promising approach for simultaneous detection (Zhu et al., 2006). Compared with the existing ELISAs, protein microarrays have the same sensitivity level and are more specific. Therefore, microarrays can be used for large-scale screening to identify specific antibodies in blood samples quickly and sensitively.

## 3 Antigen, IgM, or IgG: Which biomarker to choose?

### 3.1 Antigen

Viral shedding usually precedes the appearance of antibodies (**Figure 2A**) (Corman et al., 2016). Therefore, antigen detection is more conducive to early diagnosis than antibody detection. Several studies have demonstrated that viral antigens could be detected from various specimens during the early stage of the disease. Plasma and respiratory secretions were the first to be detected positive in the first few days after the onset of illness, and then urine and feces (Grant et al., 2003; Peiris et al., 2003; Chan et al., 2004). It was found that some antigen detection methods are more efficient or comparable to nucleic acid detection methods in the early stage of the disease. As early as 1-5 days after the onset of illness, N protein can be detected from respiratory and serum specimens with a positive rate of up to 100% (Di et al., 2005; Fujimoto et al., 2008). And the positive rate of N protein was 40% higher than that of RNA during the first 10 days of the disease course (Li et al., 2005a). In the detection of MERS-CoV, the lowest LOD of the antigen is even an order of magnitude lower than that of the nucleic acid (Yamaoka et al., 2016). The detection rate of N protein in the serum of SARS confirmed patients was 100% consistent with that of IgM (He et al., 2005a). The viral shedding of SARS-CoV-2 begins at least in the last 2-3 days of the incubation period (He et al., 2020). Therefore, detecting specific antigens such as the N protein is significant for early diagnosis and discovery of latency infections. It can be used as a complement to PCR to improve diagnostic efficiency. In the absence of qualified equipment and trained personnel, the antigen detection method can be used for preliminary screening of suspected patients, and then the nucleic acid test can be used to confirm negative results.

### 3.2 IgM and IgG

Antibody detection provides a wider time window for diagnosis. It is useful for epidemiological investigation since antibodies remain detectable longer than antigens. IgM is a valuable marker for diagnosing acute viral infections since it is the first antibody produced during an immune response and rises rapidly during the early stage of the disease course (Ehrenstein and Notley, 2010). IgG usually appears shortly after or simultaneously with IgM, reaching its peak later than IgM. What is contrary to this common sense is that in the early stage of research on SARS, it was found by several kinds of antibody detection methods that IgG became detectable earlier than or simultaneously with IgM (Chang et al., 2004; Hsueh et al., 2004b; Woo et al., 2004c). It was not until the emergence of a specially designed method, MAC-ELISA, for IgM that the mean seroconversion time for IgM was found to be 8 days after disease onset, which was 3 days earlier than that for IgG (Yu et al., 2007). It suggested that whether the seroconversion time of IgM can be revealed more accurately depends on the sensitivity of the detection method. Another study found that IgM against SARS-CoV showed higher positive rates than IgG in the early period of the disease, and this trend changed 1 month after the onset (Yang et al., 2009b). Thus, with well-designed detection methods, IgM could be a reliable serological biomarker for earlier diagnosis.

In the early stages of the disease course, the simultaneous determination of IgG and IgM helps improve the efficiency of diagnosis (Chen et al., 2004a; Woo et al., 2004a). However, over time, this advantage will not be significant. Take SARS for example, from the fourth week of onset, the efficiency of identifying real patients mainly depends on the positive rate of IgG (Chen et al., 2004a). For newly emerging infectious diseases, the detection of specific IgG in a single serum is valuable for early diagnosis or screening. Nevertheless, when the pathogen has been circulating in human beings for a long time, most patients may be re-infected. In this case, the suspected case should be confirmed by an increase of IgG titers in paired serum. Considering that IgG persists for a long time after healing, it is also suitable for retrospective epidemiological investigations and evaluation of vaccine efficacy. Few studies have focused on the diagnostic value of IgA. Wu et al. found that specific IgA appeared as early as 2-3 days after disease onset, and the simultaneous determination of IgG and IgA helped increase the detection rate (Wu et al., 2004b). However, other studies have not come to a similar conclusion (Hsueh et al., 2004b; Woo et al., 2004c). Wu et al. applied all the four structural proteins as detection antigens simultaneously, while other studies only used the N protein. Therefore, it cannot be considered that there is a contradiction between the conclusions of these studies. The diagnostic value of IgA needs to be further clarified.

## 4 Specificity and sensitivity

Sensitivity and specificity are the two most important indicators reflecting the performance of immunoassay methods. There are differences among the existing methods in these two aspects. The insufficient specificity is mainly due to the applied antigenic protein containing amino acid sequences that are highly homologous to other HCoVs, which leads to cross-reactions. It can be improved by screening and identifying specific epitopes more systematically. Two problems mainly cause the inadequacy of sensitivity. On the one hand, the LOD of the method is not low enough to detect the analytes with extremely low concentrations. On the other hand, some of the samples for antibody detection were collected from patients who had not undergone seroconversion. The former can be improved by applying some new materials and technologies with superior signal conversion and amplification performance. The latter could not be changed, but the false negative results could be rectified by supplement antigen detection since viral shedding usually occurs earlier than seroconversion.

### 4.1 Improve specificity: Screening for better epitopes and MAbs

High-quality antigens are the key to ensuring the specificity of antibody detection methods, and specific antibodies selected based on highly immunodominant epitopes are the key to ensuring the specificity of antigen detection methods (Leenaars and Hendriksen, 2005). B-cell epitope mapping is the most effective solution to achieve this goal (Malito et al., 2013). Currently, a variety of methods have been developed for B-cell epitope mapping, and they can be divided into three categories: structural methods, functional methods, and computational prediction methods (Ahmad et al., 2016). Herein, we will focus on those methods that are most often used to determine the fine amino acid sequence of the epitope.

#### 4.1.1 Structural methods

The most intuitive and effective way to find epitopes is to observe where the antigen-antibody binding occurs. It can be achieved with X-ray crystallography and cryo-electron microscopy(cryo-EM), which could display 3D structural images of the antigen-antibody complex. The use of X-ray crystallography for S1RBD of SARS-CoV led to the identification of a neutralizing antibody binding epitope that overlaps very closely with the ACE2-binding site as a potential candidate for diagnosis and treatment (Hwang et al., 2006). Barnes et al. mapped the S1A and RBD epitopes on SARS-CoV-2 S protein by cryo-EM. These two epitopes are unlikely to be affected by common mutations in different SARS-CoV-2 isolates, which is valuable for developing stable immunoassay methods (Barnes et al., 2020). However, these two methods are technically demanding and costly, which makes them unfavorable for high-throughput screening of epitopes. But they are more suitable for revealing the reaction mechanism between specific epitopes and their antibodies.

#### 4.1.2 Functional methods

Functional methods are based on the binding capacity of an antibody to antigen fragments, synthetic peptides, or recombinant antigens. The binding capacity can be evaluated by ELISA, western blot, and sometimes peptide microarray. The functional methods that enable high-throughput epitope screening mainly include surface display technology, mutagenesis, and pepscan (Potocnakova et al., 2016).

The principle of surface display technology is based on testing the binding capacity of a variety of peptides on the display platforms, such as the surface of phage, bacteria, mammalian, insect, or yeast cells, to the monoclonal antibody or antigen protein through the affinity selection method of biopanning (Potocnakova et al., 2016). It is very suitable for screening epitopes of virus antigens to meet the extreme need for precise identification of target epitopes (Gershoni et al., 2007). For instance, by panning a yeast surface display with polyclonal antisera from immunized mice and sera from convalescent SARS patients, Liang et al. identified 4 novel conformational epitopes (aa 1-69, 68-213, 212-341, and 337-422) from the N protein. These epitopes were shown to have a good potential value for clinical diagnosis (Liang et al., 2005). Kim et al. found two unique Fabs (S2A3 and S2D5) that are monospecific to the S2 subunit of the MERS-CoV S protein with a human Fab phage display library and established an ELISA system with these two Fabs (Kim et al., 2019b). The surface display technique was also combined with other mapping techniques, such as mutagenesis, to resolve the epitopes of different antigens.

Mutagenesis is a rapid epitope mapping method. It relies on the substitution of individual residue/s (hot-spot/s) that constitutes a functional epitope causes loss of antibody binding (Potocnakova et al., 2016). Combining mutagenesis with surface display technology can achieve high throughput screening with hundreds or thousands of mutated proteins. For instance, a combination of semisynthetic antibody phage display libraries and mutagenesis of recombinant SARS-CoV S fragment (aa 318-510) helped to identify an epitope in the S fragment. On this basis, six specific MAbs were selected and proved to be of diagnostic value (van den Brink et al., 2005). Similarly, Ying et al. identified three high-affinity MAbs targeting RBD of MERS-CoV S protein by combining a large phage display naïve-antibody library (containing ~1011 antibodies) and alanine mutagenesis of the MERS-CoV RBD (Ying et al., 2014). The identification process is quite rapid and could be completed within several weeks.

Pepscan is a technology that employs a panel of chemically synthesized peptides, which covers the entire amino acid sequence of the target antigen, to test their binding capacity to the desired antibody. It is ideal for identifying linear and simple conformational epitopes. Compared with the abovementioned two methods, pepscan is the most efficient and convenient approach. It is accessible without expensive equipment and expertise and can be used without the necessity of purified antigens or antibodies. Using this approach, He et al. have successively identified several immunodominant epitopes on N, S, and M proteins of SARS-CoV (He et al., 2004b; He et al., 2004c; He et al., 2005c). Amrun et al. constructed a SARS-CoV-2 peptide library of all the four structure proteins, S, E, M, and N. With the library, they identified four immunodominant epitopes on the S and N proteins by peptide-based ELISA (Amrun et al., 2020). The disadvantage of pepscan is that complex conformational epitopes which involve tertiary and/or quaternary structures are unlikely to be identified (Abbott et al., 2014).

#### 4.1.3 Computational prediction methods

The emergence of computational prediction methods, also known as in silico prediction methods, is based on the highly developed bioinformatics. These methods are prevalent because they can dramatically reduce the burden associated with epitope mapping by decreasing the list of potential epitope candidates for experimental testing (Sanchez-Trincado et al., 2017). In research on HCoVs, computational methods were primarily used for designing vaccines (Ibrahim and Kafi, 2020; Abela et al., 2021), and then were also applied to develop diagnostic methods (Lu et al., 2005b; Gao et al., 2021). Researchers usually use computational prediction methods to obtain the amino acid sequences of tentative epitopes and then use the functional methods described above to screen for immunodominant epitopes. It is much more efficient than preparing overlapping peptides, mutants, or surface display libraries spanning the entire sequence of the target protein, especially for proteins with large molecular weights like S protein.

### 4.2 Improve sensitivity: Application of new techniques

Currently, the LOD of the above methods is not low enough to detect the trace amount of analytes in real samples. Therefore, we can learn from the application of nanomaterials and microfluidic systems in other target detection methods to improve the sensitivity of immunoassay methods against HCoVs, and provide a broader idea for further research. In recent years, the emergence of advanced nanomaterials and biosensing techniques greatly facilitates the advancement of immunoassay methods in terms of sensitivity. Here we mainly introduce the most promising nanomaterials that can achieve more robust and stable signal output and digital microfluidic (DMF) systems with the potential for sensitive, rapid, and high-throughput detection.

#### 4.2.1 Metal nanoparticles act as enzyme mimics

**Figure 5:**
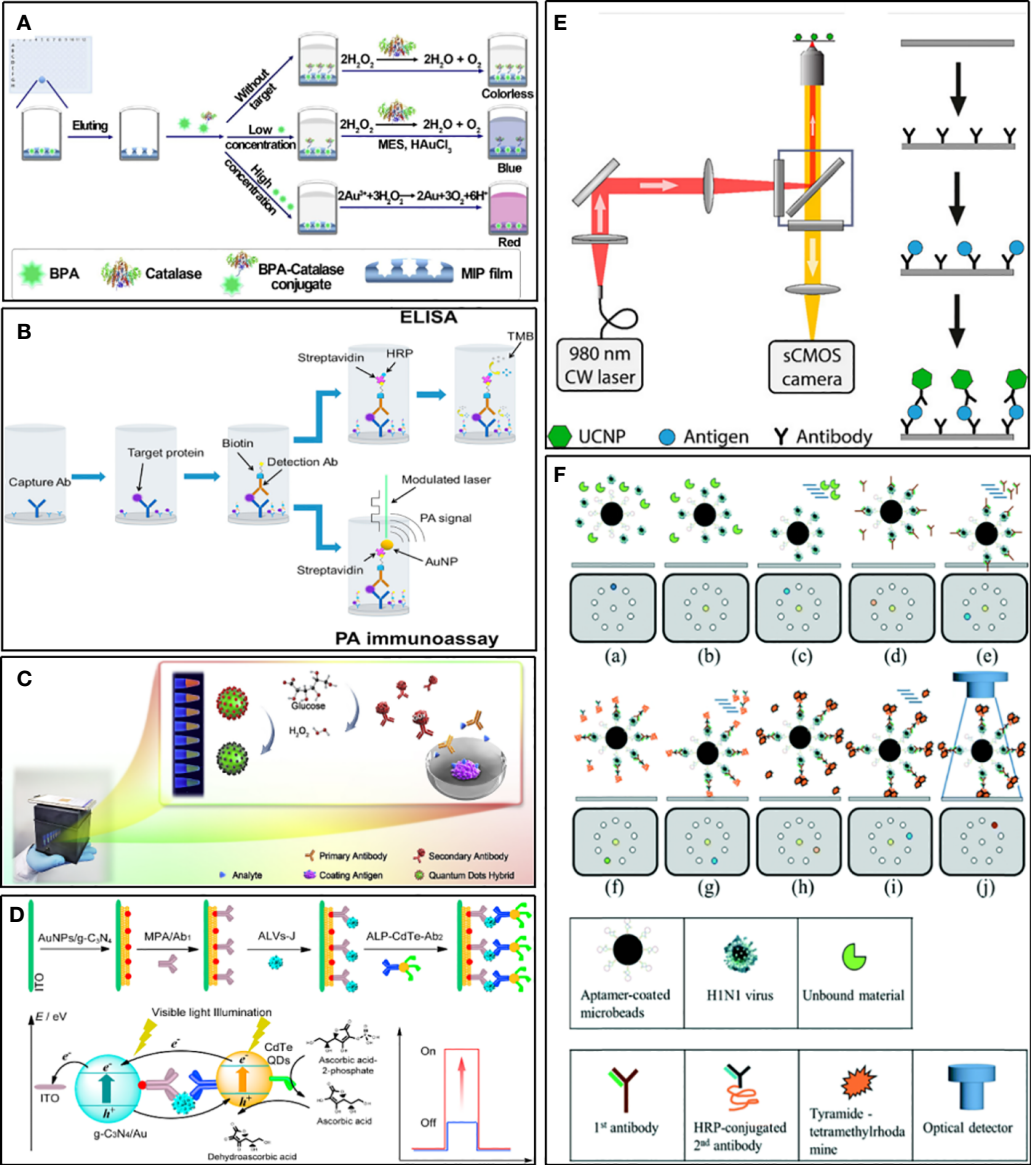
Examples of strategies for improving sensitivity. **(A)** Schematic illustration for ELISA combines MIP with catalase-mediated growth of plasma AuNPs (Jia et al., 2021). **(B)** Schematic diagram of an ELISA in which SA-HRP is replaced by an SA-bound AuNP and no TMB substrate is used (Zhao et al., 2016). **(C)** Schematic diagram of the ratiometric fluorescent sensing system integrated of a ratiometric quantum dots (QDs) hybrid and chemical redox reaction for drug residue analysis (Yu et al., 2020). **(D)** Schematic illustration for photoelectrochemical immunosensor based on AuNPs/g-C3N4 coupling with CdTe quantum dots for detection of target avian viruses (Sun et al., 2019). **(E)** Scheme of ULISA for counting single molecules of PSA (Farka et al., 2017). **(F)** Schematic diagram of influenza virus diagnostic process using magnetic beads in a structure-free digital microfluidic platform (Lu et al., 2020).

In conventional ELISA, the key factors that limit the sensitivity of the assay are mainly the instability of natural enzyme labels and the weak generation of output signal values (Prasse et al., 2014). Metal nanoparticles (MNPs), such as gold nanoparticles, platinum nanoparticles, and silver nanoparticles (Loynachan et al., 2018; Mohamad et al., 2019), not only have stable and efficient peroxidase-like activity and play an important role in immunosensors, but also be used as colorimetric substrates in ELISA. Moreover, their color changes are more significant based on the principle of local surface plasmon resonance (LSPR). Jia et al. (Jia et al., 2021) established a sensitive ELISA for bisphenol A (BPA). It combines molecularly imprinted polymer (MIP) membrane, used as a biomimetic antibody, with catalase-mediated growth of plasma AuNPs. In quantitative analysis, LOD is 6.20 pg/ml (**Figure 5A**). Zhao et al. applied AuNPs as signaling reports to detect human interleukin-8 chemokine (Zhao et al., 2016). Because of LSPR, AuNPs can interact strongly with intensity modulated laser excitation and produce strong plasma enhanced photoacoustic effect. The LOD of this method is about 143 times higher than that of gold standard ELISA, which decreases from 23 pg/mL to 0.16pg/mL (**Figure 5B**). Of course, many other metal nanoparticles are also used to mimic enzymes and chromogenic substrates, which give considerable inspiration to the sensitive detection of HCoVs.

#### 4.2.2 Quantum dots and upconversion nanoparticles for luminescence

Quantum dots (QDs), a kind of fluorescent inorganic semiconductor nanoparticles, are superior alternatives to traditional fluorescent probes for their merits such as broad excitation spectra, narrow emission spectra, easy to modify, size-tunable fluorescence emission, and good fluorescent stability (Resch-Genger et al., 2008; Dong et al., 2014). QDs hybrids, such as CdSe@SiO2@CdTe, could display multiple color responses changing with the different concentrations of analyte, which makes them useful for straightforward visual detection with high sensitivity (**Figure 5C**) (Yu et al., 2020). Compared with conventional colorimetric ELISA, the ELISA using QDs as the signal generator can realize visual measurement with the signal amplified by about 2 orders of magnitude (Wu et al., 2015). When square-wave voltammetry (SWV) was used for signal measurement, the sensitivity increased by 100 times. Using dendrimers to amplify the signal of QDs further, and combine it with a solid surface immunosensor, the LOD at the level of fg/mL could be achieved (Babamiri et al., 2018). Given the versatile photochemical properties of QDs, they are very suitable for use as photoactive species to fabricate photoelectrochemical biosensors. This kind of immunosensor has been applied to detect virus antigens, such as subgroup Javian leukosis viruses (ALV-J) and hepatitis B virus, which showed high sensitivity (**Figure 5D**) (Tan et al., 2017; Sun et al., 2019).

Upconversion nanoparticles (UCNP) are a new generation of nanoparticles. It can exhibit conversion from near infrared (NIR) to shorter NIR, visible light, or UV (Haase and Schäfer, 2011). The unique anti-Stokes optical property makes it useful for realizing highly sensitive background-free measurements with minimal autofluorescence and scattered light of the surrounding matrix (Wen et al., 2018). As a novel fluorescent label, UCNP is used to develop various immunological assay methods, such as ELISA, lCT, and protein microarrays (van Dam et al., 2013; Farka et al., 2017; Gong et al., 2019). Some of these researches have been compared with traditional methods and showed the superiority of using UCNP as labels. An ICT for detecting active schistosomiasis could perform as good as ELISA in sensitivity and specificity, and was found to be superior concerning the speed and simplicity of use (van Dam et al., 2013). Moreover, an ELISA for the detection of the cancer marker prostate-specific antigen (PSA) was about ten times more sensitive than commercial ELISAs and could count single molecules (**Figure 5E**) (Farka et al., 2017).

#### 4.2.3 Digital microfluidics for trace detection

DMF is a new method for working on open surfaces and using droplet control. Compared to traditional continuous-flow microfluidics, digital microfluidics have the following characteristics: lower sample and reagent consumption, each droplet running independently, and the ability of each droplet to act as a separate reaction chamber (Zhang and Nguyen, 2017). Droplet digital ELISA is developed based on DMF technologies as a simple method to detect protein with low concentrations. It is more sensitive than traditional ELISA (Cohen et al., 2020). Therefore, it has a strong advantage in improving the performance of viruses and potential biomarker detection. For example, Lu and his co-workers developed a magnetic digital microfluidic platform using an ELISA-like assay to detect Influenza A (**Figure 5F**) (Lu et al., 2020). Magnetic microbeads coated with H1N1-specific aptamers were used to actuate the microfluid. The H1N1-specific aptamers were used to capture target viruses. The HRP-conjugated secondary antibody was used as a probe to catalyze substrates to generate fluorescent signals. The fluorescent signal was amplified by tyramide-tetramethylrhodamine (TTMR) and was used to quantify the magnetic complexes. The principle of this process has no different from ELISA, but the reaction system based on a small number of molecules makes it much more sensitive than traditional methods. The LOD was 1000-fold lower than the conventional immunoassays, and the entire detection process took only 40 minutes, which proved the great potential of digital microfluidics technology in rapid diagnosis.

## 5 Conclusion and implication for SARS-CoV-2

During and within a few years after the outbreak of SARS and MERS, a good deal of diagnostic immunoassay methods has been established. At the same time, the detection methods for the other four HCoVs have also been developed. Since the COVID-19 outbreak, immunoassay methods for SARS-CoV-2 have also mushroomed. Given similar structures and immune responses of HCoVs, the summarization of these existing methods showed important implications for the development of novel detection methods for SARS-CoV-2 and other unknown coronaviruses that may emerge in the future.

Based on this review, immunoassays should use recombinant proteins rather than virions as antigen reagents to facilitate standardization and popularization. Among the existing methods, recombinant N protein is employed most frequently and its diagnostic value has been comprehensively revealed. Methods based on full-length N proteins usually have at least moderate performance. An ideal recombinant protein should be one with the conserved amino acid sequences, common to all HCoVs, deleted as completely as possible and retain as many immunoreactive epitopes as possible.

Immunoassay methods based on such recombinant proteins usually achieve high levels of sensitivity and specificity. For the N protein of HCoVs, truncating part of the amino acid at the N-terminus seems to be an effective method. The application frequency of the S protein is second only to the N protein. Its diagnostic value has been partially clarified. Methods based on the S1 subunit generally exhibit good performance, but the exact amino acid sequences that contain the best epitopes remain to be further explored. In order to make S protein-based methods show good performance, the S protein should be produced by the eukaryotic expression system, which can ensure the correct expression of its conformational epitopes. Because the immune system first produces antibodies against conformational epitopes when a virus infection occurs, the detection of antibodies against conformational N or S protein can make sense for an earlier diagnosis. In addition to the N protein and the S protein, the M protein and some non-structural proteins also seem to have particular diagnostic value, which needs further study. When exploring the diagnostic value of these antigenic proteins, epitope mapping is a robust approach. In the process of developing new methods, it is suggested to establish methods based on multiple antigenic proteins to achieve better performance. The optimal combination of antigens is worthy of an in-depth study.

This review mainly summarizes the four mainstream immunoassay methods. Based on these methods, new sensor technologies could be used to improve their performance to achieve more sensitive and faster detection. Among these methods, ELISA is the most useful immunoassay for high throughput detections. The advantage of ELISA is that a mixture of multiple antigens or antibodies can be used as a coating reagent to achieve a sufficient capture of the analytes. Based on conventional ELISA, the derived methods such as CLEIA, MMIA, and microprotein array can achieve more sensitive and even multiple detections. These detection methods need to be implemented in a laboratory with specific equipment. ICT is the best approach for POCT, but its sensitivity is often less than ideal. It should be mainly due to the insufficient signal amplification capability of the commonly used colloidal gold label. Newly developed nanomaterials, such as QDs and UCNPs can be considered utilized for the sensitive determination of SARS-CoV-2. IFA and WB are suitable for the confirmatory test. IFA directly uses eukaryotic cells as the provider and carrier of the antigen reagent, which could ensure the most proper expression of conformational epitopes. It is useful to ensure the specificity of the method. WB can simultaneously detect antibodies against multiple antigens in one test, providing more information for diagnosis than other methods. The traditional WB requires skilled laboratory staff, while the line immunoassay modified from WB has the potential to be commercialized for its easier operation. In addition, immunosensors could be further developed into POCT methods considering their sensitivity and portability. However, there is still a long way to go to achieve widespread application for its current high economic cost. Generally, ELISA and ICT are the most valuable for commercialization. Their customers are different. ELISA is mainly demanded by hospitals and public health institutions. ICT is mainly used to meet the needs of self-testing and diagnosis in resource-limited areas. Together, they form integral parts of epidemic control. Although the commercialization of immunosensor is still difficult, the existing problems will be eventually solved with the maturity of related technologies. For any specific method, it must be rigorously evaluated based on real samples before it can be commercialized.

In the previous development of methods for HCoVs, the lack of well-characterized clinical samples prevented many methods from being validated. In order to develop more reliable detection methods for SARS-CoV-2, serum samples of confirmed patients from different populations and stages in the course of the disease should be systematically collected. The control group should include people who are or have been infected with the other four non-severe acute respiratory syndrome HCoVs, as well as healthy people. The former is particularly important for confirming the specificity of the method because over 70% of the general public has seroconverted towards all four non-severe acute respiratory syndromes HCoVs, which mainly contribute to the cross-reactivity.

Since SARS-CoV-2 is likely to circulate in humans for a long time, like the influenza viruses, reliable and efficient immunoassay kits against SARS-CoV-2 will be demanded in large quantities for a long time. We hope that this review could provide a reference for the further research.

## Author contributions

All authors listed have made a substantial, direct and intellectual contribution to the work, and approved it for publication.
